# Beyond a seat at the table: Embedding lived experience in mental health ethics committees

**DOI:** 10.1371/journal.pmen.0000545

**Published:** 2026-02-13

**Authors:** Parth Sharma, Aiswarya S., Harikeerthan Raghuram, Anant Bhan

**Affiliations:** iHEAR, Sangath, Bhopal, India; PLOS: Public Library of Science, UNITED KINGDOM OF GREAT BRITAIN AND NORTHERN IRELAND

## Ethics of lived experience inclusion

It is worth enquiring, when attempts at diversity and equity further epistemic injustice, what true purpose do they serve?

Recent years have seen a notable shift towards Lived Experience inclusion in academia and research, especially with the visible international ethics nomenclature shifting towards community engagement, patient and public involvement and service user representation [1]. Evidenced from the revised Declaration of Helsinki guidelines, commitments by funding organizations such as Wellcome Trust, scientific journals like The Lancet group and PLOS and the 2023 WHO framework for Lived Experience Inclusion, Lived Experience is no longer a foreign and contested topic, but now a perquisite for many institutions [2–6].

While the larger ethics ecosystem now recognizes the importance of inclusion, the design and more importantly the execution of these efforts routinely undermine the very commitment of equity they claim to uphold. Over the years People with Lived Experience have experienced tokenistic inclusion of minority identities, extractive research practices, a lack of fair compensation and outright use of dehumanizing language. Many have also written extensively imagining possibilities for a future beyond such appalling narratives [7]. While many of these appalling accounts exist, they do so predominantly in informal settings, community zines, and non-academic communications as a method of resisting further discrimination. It is rare to hear such accounts in sanitized spaces of academia, however; it doesn’t deny their existence and that of systems that allow those with institutional power to continue to replicate the oppressive fabric of extractive research [8].

Today, laypersons from communities are represented in Institutional Ethics Committees [9]. Although it is necessary to problematize this language of *laypersons*. The insidious linguistic marker of “Lay” along with the numerous contradictions over its use in research often represent individuals that engage in research from outside the academic industrial complex. It is understood as those that do not have *expertise* in the subject matter being researched and are often the voices of those *being researched upon.* Besides the often-glaring omission of intersectionality within the representation of *laypersons*, the larger question remains, is having one non-expert or very few at best on the committee enough?

Ethics Committees remain a crucial site for accountability within decision making in Mental Health Science and represent a set of voices that are expected to work to ensure utmost rigor and ethics, especially around human participation [10]. These diverse voices through the committee as a collective can guide the decision-making process from the time of study design, to study implementation, analysis and reporting post completion of the research. Ethics Committees are entrusted with safeguarding not only the international and domestic guidelines on human participation, but importantly the larger welfare and dignity of research participants [10].

Their [Ethics Committees’] role as an institution of importance cannot be overstated; however, their design and operational infrastructure are deep-rooted in inequities that require critical reflection. Ethics Committees can, insofar, determine research guidelines; however, they often struggle with monitoring study conduct on an ongoing basis, and stopping studies from taking place that do not adhere to said guidelines. Here they play the role of prospective gatekeepers. Within the ever-growing ecosystem of Lived Experience, it becomes important to note where the role of Ethics Committees ends, and that of the publisher begins. Publishers run the risk of representing sites that act as retroactive gatekeepers of knowledge production demanding adherence to local ethics yet can routinely choose to make exceptions and give legitimacy to research that perpetuates Lived Experience erasure.

## Inclusion requires infrastructure

At first glance this growing movement for inclusion (having a seat at the table) looks well intentioned and progressive, yet the process of inclusion is not inherently ethical. Inclusion centered around providing *a seat at the table,* risks creating an archetype of a singular person with Lived Experience that represents several heterogenous and often intersecting movements. Such a reductive understanding views Lived Experience as a checkbox on an ethics checklist as opposed to a meaningful process of engagement. Furthermore, singular representation on Ethics Committees becomes symbolic and extractive, where Lived Experience remains a tool to validate preexisting decisions to meet superficial diversity and bureaucratic requirements, akin to the increasingly visible processes of participatory assimilation [11]. This is true whether it is representation on Ethics Committees or in research leadership teams.

Such tokenism in decision making roles also requires extensive labor from the singular person with Lived Experience to adapt and work in rooms that were not designed for their participation as opposed to institutional safeguarding where mechanisms for continuing support exist. Without structural support for meaningful engagement, these Ethics Committees run the risk of reinforcing epistemic hierarchies and perpetuating and becoming sites of structural violence and epistemic injustice.

A recurring response to critiques of tokensim in Lived Experience Inclusion arises from the perception of resource scarcity. “*The best was done with what was given*” phenomenon actively justifies marginal inclusion and the lack of structural support which is evidenced through nominal honorarium, lone consultative roles and a lack of expenditure for skill and capacity enhancement. This also speaks to a lack of co-design from the conceptualization stage [12]. Expenditure required to build and sustain meaningful engagement of Lived Experience is not deemed important enough to budget, while bylines include session honorariums and minimal compensation based on arbitrary industry standards. For many people with Lived Experience, little to no safeguards exist in such scenarios. Consultation based work agreements do not include mechanisms of redressal, emergency funds or institutional support, and fair remuneration often becomes a long and tedious process to receive. To give the lie to this myth of resource scarcity, enough resources exist to bring lived experience to *a temporary seat at the decision-making table.*

Ethical inclusion demands investment and infrastructure.

## Embedding lived experience: where do we go from here?

To embed intersectional lived experience, it becomes imperative that we reconceptualize inclusion beyond token representation and create pathways for meaningful power-sharing, equitable remuneration, institutional safeguarding, and democratic governance structures.

Within Ethics Committees, this asks for the rejection of the procedural format of ethics, one that starts with a paternalistic and often deficit-based approach [13]. An approach which rests on the assumption that People with Lived Experience are inherently without agency and are vulnerable and fragile and can only opine on limited aspects of the review process such as ‘informed consent form’ language or ‘community engagement’ outreach plans. Not only does this de-value the expertise within Lived Experience; but it also routinely sanitizes “ethics” purely as an academic exercise to be conducted within the boundaries of the ethics committee.

Mental health research is inherently political. From funding priorities often set in the Global North, to grant conceptualization and study design conducted in silos, and the growing evidence base on best practices; epistemic agency is rarely afforded to those with multiple marginalized identities and lived experience who do not reside in ivory towers [14]. The ethics of this research, then, remain inherently political as well, and it becomes essential that the Ethics Committees reflect this experience. By working with local and grassroots community-based organizations and mental health lived experience collectives, institutions hosting Ethics Committees can work towards centering intersectional lived experience and intentionally strengthen the infrastructure through which community wisdom can be shared and sustained– in the community itself. This not only calls for a critical reflection on where we situate ethics in research, but also acknowledges the heterogeneity of Survivor, Mad Pride, Disability Justice, Service User, and Lived Experience voices as distinct and equally important forms of expertise [14].

While attempts at inclusion bring with them a series of risks and challenges, it is prudent to admit that they also pose a unique opportunity for institutions, researchers and funders to collaborate with people with lived experience. Done intentionally towards the goal of ethical inclusion; this collaboration can help move beyond creating *a seat at the table*, and towards a process of meaningful co-design.

## References

[1] Fischer L a u r a, et al. Breaking boxes: announcing the Lancet Psychiatry Commission on Lived Experience in Mental Health Research. The Lancet Psychiatry. 2023;11(6):406–8. 10.1016/S2215-0366(24)00137-8 38760106

[2] Parums DV. Editorial: The 2024 Revision of the Declaration of Helsinki and its Continued Role as a Code of Ethics to Guide Medical Research. Med Sci Monit. 2024;30:e947428. doi: 10.12659/MSM.947428 39616449 PMC11619173

[3] Embedding lived experience in mental health research. https://wellcome.org/research-funding/guidance/prepare-to-apply/embedding-lived-experience-expertise-mental-health-research

[4] Lived Experience Commission. Home. LE Commission. https://www.livedexperiencecommission.org/. 2023.

[5] Montague-Cardoso K, Sunkel C, Burgess RA. PLOS Mental Health: Elevating the voices of lived experience to combat structural barriers and improve mental health globally. PLOS Ment Health. 2024;1(1):e0000053. doi: 10.1371/journal.pmen.0000053 PMC12798434 41661794

[6] Meaningful Engagement of People with Lived Experience. https://www.who.int/groups/gcm/meaningful-engagement-of-people-with-lived-experience. 2022.

[7] Gatera G, Singh S. Beyond tokenism: the influence of lived experience and its future possibilities in mental health science. Nat Mental Health. 2023;1(3):151–2. doi: 10.1038/s44220-023-00027-x

[8] Anderson J, Spandler H. Mad Zine pedagogy: using zines in critical mental health learning and education. Social Work Education. 2025;1–19. doi: 10.1080/02615479.2025.2469586

[9] Ganesh D, Kalikar MV. Role of lay person in ethics committee: Bridging expertise and public trust. Perspect Clin Res. 2025;16(2):99–101. doi: 10.4103/picr.picr_232_24 40322471 PMC12048089

[10] Mehta P, Zimba O, Gasparyan AY, Seiil B, Yessirkepov M. Ethics Committees: Structure, Roles, and Issues. J Korean Med Sci. 2023;38(25):e198. doi: 10.3346/jkms.2023.38.e198 37365729 PMC10293659

[11] Veldmeijer L, van Os J. “They talk the talk but don’t walk the walk”. Introducing the concept of participatory assimilation in mental health care innovation. Psychosis. 2025;1–12. doi: 10.1080/17522439.2025.2560852

[12] Barker G. The myth of scarcity. Democracy Network. https://democracynetwork.org.uk/the-myth-of-scarcity/. 2024.

[13] Dembele L, Nathan S, Carter A, Costello J, Hodgins M, Singh R, et al. Researching With Lived Experience: A Shared Critical Reflection Between Co-Researchers. International Journal of Qualitative Methods. 2024;23. doi: 10.1177/16094069241257945

[14] Sharma P. Reparations not remuneration: Redefining the future of lived experience. PLOS Ment Health. 2025;2(11):e0000495. doi: 10.1371/journal.pmen.0000495 PMC12798572 41662111

